# Automated Geo/Co-Registration of Multi-Temporal Very-High-Resolution Imagery

**DOI:** 10.3390/s18051599

**Published:** 2018-05-17

**Authors:** Youkyung Han, Jaehong Oh

**Affiliations:** 1 School of Convergence & Fusion System Engineering, Kyungpook National University, Sangju 37224, Korea; han602@knu.ac.kr; 2 Department of Civil Engineering, Korea Maritime and Ocean University, Busan 49112, Korea

**Keywords:** georegistration, co-registration, Kompsat-3, registration noise

## Abstract

For time-series analysis using very-high-resolution (VHR) multi-temporal satellite images, both accurate georegistration to the map coordinates and subpixel-level co-registration among the images should be conducted. However, applying well-known matching methods, such as scale-invariant feature transform and speeded up robust features for VHR multi-temporal images, has limitations. First, they cannot be used for matching an optical image to heterogeneous non-optical data for georegistration. Second, they produce a local misalignment induced by differences in acquisition conditions, such as acquisition platform stability, the sensor’s off-nadir angle, and relief displacement of the considered scene. Therefore, this study addresses the problem by proposing an automated geo/co-registration framework for full-scene multi-temporal images acquired from a VHR optical satellite sensor. The proposed method comprises two primary steps: (1) a global georegistration process, followed by (2) a fine co-registration process. During the first step, two-dimensional multi-temporal satellite images are matched to three-dimensional topographic maps to assign the map coordinates. During the second step, a local analysis of registration noise pixels extracted between the multi-temporal images that have been mapped to the map coordinates is conducted to extract a large number of well-distributed corresponding points (CPs). The CPs are finally used to construct a non-rigid transformation function that enables minimization of the local misalignment existing among the images. Experiments conducted on five Kompsat-3 full scenes confirmed the effectiveness of the proposed framework, showing that the georegistration performance resulted in an approximately pixel-level accuracy for most of the scenes, and the co-registration performance further improved the results among all combinations of the georegistered Kompsat-3 image pairs by increasing the calculated cross-correlation values.

## 1. Introduction

Owing to the frequent accessibility of very-high-resolution (VHR) satellite images, time-series analysis using VHR multi-temporal images has been conducted for a wide range of remote-sensing applications [1,2,3,4,5,6]. For the successful applications using VHR multi-temporal satellite images, accurate georegistration to the map coordinates in approximately pixel-level accuracy should be conducted [7]. Most VHR satellite images provide rational polynomial coefficients (RPCs) that represent the ground-to-image geometry, allowing for photogrammetric processing without requiring a physical sensor model. According to the specifications of the state-of-the-art VHR satellites, the level of RPC accuracy is ±10 pixels at a circular error of 90% (CE90) [8]. To achieve higher accuracy, such as up to a one-pixel level, the bias-compensation of RPCs is often manually conducted utilizing global navigation satellite system (GNSS) survey or accurate reference data. To automate the bias-compensation procedure, it requires corresponding points (CPs) between images together with accurate extraction of three-dimensional (3D) ground control points from reference data. There are many studies focusing on extracting CPs to compensate for the bias involved in the RPCs [8,9,10,11,12,13,14].

In the literature, there are two types of methods for the extraction of CPs: area-based and feature-based [15]. The area-based method uses windows of a predefined size at each image and identifies a peak of similarity between the windows for extracting the CPs. It works well in a situation in which images do not have severe geometric differences in terms of scale, rotation, and translation. In this situation, a search space of the sliding window to identify the similarity peak associated with the CPs can be geometrically limited. The area-based method is likely to fail when the multi-temporal images show significant distortions or large geometric differences. The feature-based method extracts CPs based on representative points on the images; for example, dominant features, line intersections, or centroid pixels of close-boundary regions (i.e., segments). The extracted representative points between images are matched to one another using various descriptors or similarity measures, along with spatial relationships. The feature-based method can be applied even in the case that the images have significant distortions and geometric differences. Thus, the feature-based method is known to be more effective when working with VHR multi-temporal images when compared with the area-based method [16,17]. However, applying well-known feature-based matching methods, such as scale-invariant feature transform (SIFT) [18] and speeded up robust features (SURF) [19], for VHR multi-temporal images also has limitations, because they cannot be used for matching an optical image to heterogeneous non-optical data for georegistration. Moreover, it is not necessary to identify the CPs from the entire image even if a spatial relation between the images (e.g., RPCs or rough transformation coefficients) is known. A limited search space for finding CPs enables their successful extraction. Furthermore, linear features can be an option to perform georegistration, instead of using point-based features. For example, Oh et al. [20] proposed a relative edge cross-correlation (RECC) to extract corresponding linear features between VHR satellite images and airborne lidar data, in order to obtain 3D coordinates of the features exploited for the bias-compensation of RPCs.

Although georegistration based on the bias-compensation of RPCs is conducted, VHR multi-temporal images still show a local misalignment because of dissimilarities in the image acquisition conditions, such as the stability of the acquisition platform, the off-nadir angle of the sensor, and the relief displacement of the considered scene [21]. To solve these problems, a large number of CPs in regions where local misalignments are severe should be detected, after which non-rigid transformation models to mitigate the local distortions should be used [22,23,24,25]. However, obtaining evenly distributed CPs over the entire image is difficult, and thus these models cannot completely solve the problem. Some studies have focused on fine co-registration while minimizing the local misalignment occurring among the georegistered multi-temporal images. An intuitive approach first extracts a few CPs, but ones that are reliable, to coarsely co-register the images, and then achieves a fine co-registration with a large number of CPs extracted using a search space limited from the coarse co-registration step [22,24,25]. In some studies, residual misregistration information occurring at a local level even after co-registration, also referred to as registration noise (RN), is extracted and directly employed in the design of an approach for fine co-registration of VHR multi-temporal images [21,26]. In the case of fine co-registration of VHR images acquired from an unmanned aerial vehicle (UAV) to generate an accurate orthophoto, navigation information derived from GNSS-equipped UAVs is used for constraining the search space to extract a large number of well-distributed CPs [27,28].

Via a related literature review, for successful time-series applications using VHR multi-temporal images, it is important to register them onto the map coordinates (which is related to global georegistration) with a subpixel-level accuracy (which is related to fine co-registration). Therefore, this paper proposes an automated geo/co-registration framework for full-scene images acquired from a VHR optical satellite sensor. The proposed method consists of two primary steps: (1) global georegistration and (2) fine co-registration. During the first step, two-dimensional (2D) multi-temporal satellite images are matched to 3D topographic maps by applying the RECC to accurately assign the map coordinates. Then, dominant RN pixels are extracted between the georegistered images, after which a local analysis of the RN, termed registration noise-based cross-correlation (RNCC), is conducted for extracting a large number of well-distributed CPs. The CPs are finally used to construct a non-rigid transformation function that enables minimization of the local misalignment existing among the images. Experiments conducted on five Kompsat-3 full-scene images confirmed the effectiveness of the proposed framework.

The remainder of this paper is organized as follows. Section 2 introduces the proposed geo/co-registration technique in detail. Section 3 describes the datasets constructed and illustrates the experimental results. Section 4 presents a summary and conclusion.

## 2. Methods

**Figure 1:**
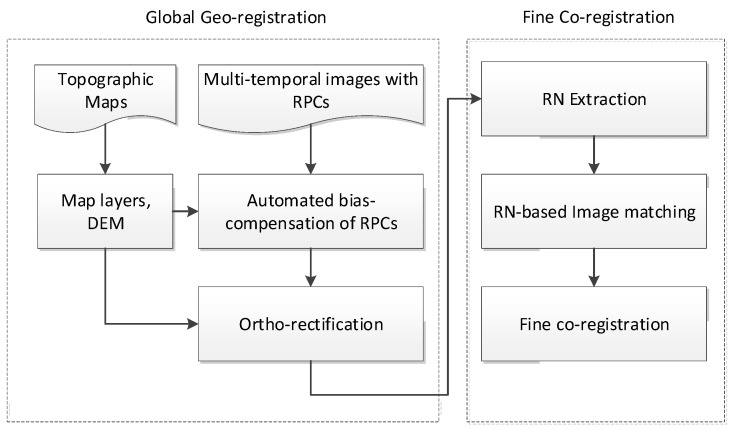
Flowchart of the automated geo/co-registration approach for very-high-resolution (VHR) time-series data set. RPCs—rational polynomial coefficients; DEM—digital elevation model; RN—registration noise.

The proposed automated geo/co-registration approach consists of two main steps: global georegistration and fine co-registration. During the global georegistration step, layers and digital elevation models (DEMs) are extracted from topographic maps to be used for the bias-compensation of the RPCs. The updated RPCs are then applied to ortho-rectify the VHR satellite images onto the map coordinates. During the fine co-registration step, the dominant RN pixels between the ortho-rectified images are extracted, which are used to identify a large number of well-distributed CPs over the entire overlapping region between the images. A non-linear transformation function estimated by the CPs is finally exploited to apply the fine co-registration. Figure 1 presents a flowchart of the proposed approach. A detailed explanation of each step will be provided in the following sub-sections.

### 2.1. Global Georegistration

Unlike medium- and low-resolution satellite images, in which 2D georegistration is largely used, VHR images require 3D control points for accurate georegistration via a bias-compensation of the RPCs. Although state-of-the-art VHR satellites show unprecedented georegistration accuracy without any ground control points, reference data are still required for better accuracy up to a one-pixel level. Among many reference data, such as LiDAR and aerial images, we utilized topographic maps that contain topographic and man-made features, including streets and buildings, because they are well developed in large-scale detail and periodically updated by government agencies.

**Figure 2:**
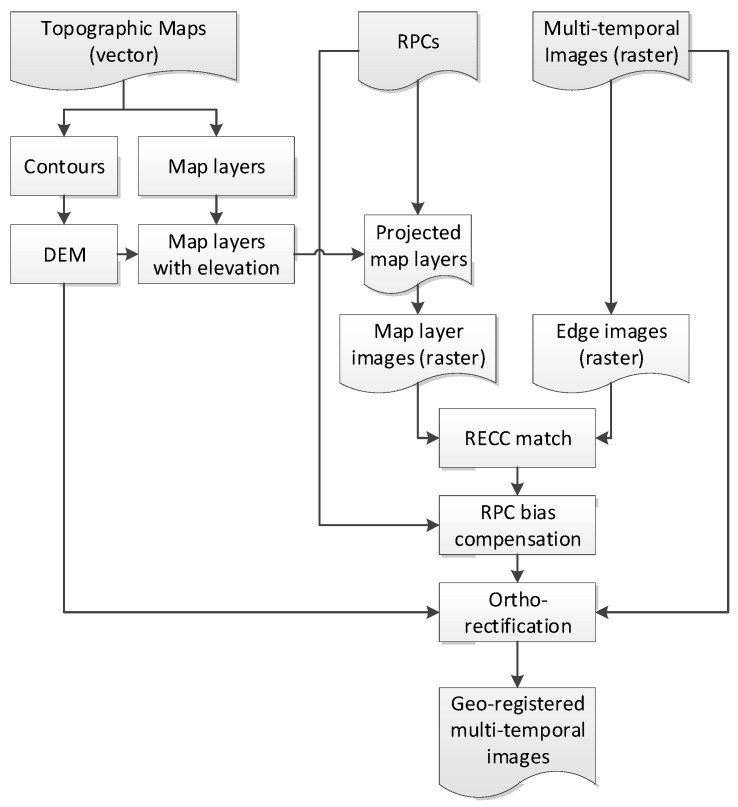
Detailed flowchart of global georegistration process. RECC—relative edge cross-correlation.

One issue is the identification of corresponding locations between VHR images and topographic maps, which is labor-intensive and time-consuming. To automate this task, edge information is extracted from the VHR images and used to match to linear features in maps. Figure 2 shows a flowchart in which multi-temporal VHR images with RPCs and topographic maps are provided. Contours and elevation points in maps enable the creation of DEMs and update elevation information in the selected layers of roads, land uses, and buildings that were extracted from the topographic maps. The map layers are projected into the image space and rasterized using the given RPCs that need to be bias-compensated. The VHR edge image is generated by applying Canny edge operator to the VHR image. The rasterized map layer is matched to the edge of the VHR image using RECC and the bias over the entire image can be estimated. Finally, the provided RPCs are compensated and used for ortho-rectification of the VHR images. Note that in natural or semi-natural environments where most edges are fuzzy, the applicability can be lower.

Edge matching is conducted using RECC and is expressed in Equation (1) [8]. It computes the number of overlapping edge pixels between the rasterized map layer and the VHR image and divides it by the total number of the edge pixels in both data. As an RECC value is relative depending on the density of edge pixels, $CV_{4}$ is computed as Equation (2) to select reliable matching points. The point with a $CV_{4}$ less than an established threshold is considered a reliable match.

(1) $$\mathrm{RECC}=\frac{\sum_{i=1}^{w}\sum_{j=1}^{w}\left(L_{ij}\times R_{ij}\right)}{\sum_{i=1}^{w}\sum_{j=1}^{w}L_{ij}+\sum_{i=1}^{w}\sum_{j=1}^{w}R_{ij}}$$

(2) $$CV_{4}=\frac{\sum_{i=1}^{4}\sqrt{{\left(r_{max}-r_{i}\right)}^{2}+{\left(c_{max}-c_{i}\right)}^{2}}}{4}$$

where L is a sliding window of the rasterized map layer (size: w×w) and R is a VHR edge image (size: (w+buffer)×(w+buffer)). $CV_{4}$ is the average distance in pixels from the maximum $\left(r_{max},c_{max}\right)$ to the four largest RECC pixels.

The bias-compensated RPCs model is expressed as Equation (3) by incorporating shift and drift parameters ($A_{0},A_{1},\ldots ,B_{2}$) into the RPC equation. The parameters are estimated using the RECC matching results over the entire VHR image region as follows:

(3) $$l+A_{0}+A_{1}l+A_{2}s=\frac{a^{T}u}{b^{T}u}L_{S}+L_{O}\;s+B_{0}+B_{1}l+B_{2}s=\frac{c^{T}u}{d^{T}u}S_{S}+S_{O}$$

with

$$u={\left[1\;V\;U\;W\;VU\;VW\;UW\;V^{2}\;U^{2}\;W^{2}\;UWV\;V^{3}\;VU^{2}\;VW^{2}\;V^{2}U\;U^{3}\;UW^{2}\;V^{2}W\;U^{2}W\;W^{3}\right]}^{T}\;U=\frac{\phi -\phi_{O}}{\phi_{S}},\;V=\frac{\lambda -\lambda_{O}}{\lambda_{S}},\;W=\frac{h-h_{O}}{h_{S}}\;a={\left[a_{1}\;a_{2}\;\ldots \;a_{20}\right]}^{T},\;b={\left[1\;b_{2}\;\ldots \;b_{20}\right]}^{T},\;c={\left[c_{1}\;c_{2}\ldots c_{20}\right]}^{T},\;d={\left[1\;d_{2}\ldots d_{20}\right]}^{T}$$

where a,b,c,d are RPC coefficients; (ϕ,λ,h) are the geodetic latitude, longitude, and ellipsoidal height, respectively; (l,s) are the image row and column coordinates, respectively; (U,V,W) are the normalized ground coordinates; ($\phi_{O},\lambda_{O},h_{O},S_{O},L_{O}$) and ($\phi_{S},\lambda_{S},h_{S},S_{S},L_{S}$) are the offset and scale factors, respectively, for the latitude, longitude, height, column, and row; and $A_{0},A_{1},\ldots ,B_{2}$ are for an affine transformation.

### 2.2. RN-Based Fine Co-Registration

The globally georegistered multi-temporal satellite images still have local misalignments between the data. Fine co-registration should thus be applied to minimize such misalignments for achieving accurate results of time-series analysis. To directly address this problem, as previously mentioned, RN is extracted to be exploited for applying the fine co-registration.

#### 2.2.1. RN Extraction

RN mainly occurs along boundaries between objects, meaning that RN has a high probability of being detected in high-frequency content regions. Therefore, we use an edge magnitude image constructed by the difference in the Gaussian (DoG) filter to extract the dominant RN pixels [29]. Let us assume that $X^{h}$ is the edge magnitude image, which is defined as Equation (4) as follows:

(4) $$X^{h}=\frac{\sum_{b=1}^{B}\left(G_{k\sigma }\left(X_{b}\right)-G_{\sigma }\left(X_{b}\right)\right)}{B}$$

where $G_{\sigma }$ is the Gaussian filter with standard deviation σ and k is a constant multiplicative factor. The values of σ and k control the thickness and smoothness of the edges in the image. B is the number of corresponding spectral bands between the images.

There are two conditions to satisfy when extracting the RN on the edge magnitude images. The first condition is that the RN occurs in the surroundings of the dominant edges in both images. Pixels along and near the dominant edges have larger edge magnitude values, and the values decrease moving toward homogeneous regions. Thus, candidate RN pixels are those having high edge magnitude in both images, as in Equation (5) as follows:

(5) $${\mathrm{RN}}_{1}\in \{\begin{array}{cc} 1, & \mathrm{if}\;min\left(\left|X_{ref}^{h}\right|,\alpha \left|X_{sen}^{h}\right|\right)\geq T_{1}\\ 0, & \mathrm{otherwise} \end{array}$$

where α is a normalization parameter that controls the balance of the edge magnitude between the two images. It can be derived as $\alpha =\sigma \left(X_{ref}^{h}\right)/\sigma \left(X_{sen}^{h}\right)$, where $\sigma \left(X_{ref}^{h}\right)$ and $\sigma \left(X_{sen}^{h}\right)$ are standard deviations of each edge magnitude image and $T_{1}$ is a threshold value.

In the case that object boundaries have strong edge magnitude in both images, candidate RN pixels, estimated using Equation (5), can be (i) precisely aligned border regions of objects or (ii) RN pixels. To discriminate between the two cases, the difference between the two edge magnitude images $X_{ref}^{h}$ and $X_{sen}^{h}$ is considered. The edge magnitude difference tends to be small for precisely aligned edges and increases as the level of misalignment increases. This is because high edge magnitude regions (i.e., dominant edges) in one image are compared with lower edge magnitude regions (i.e., regions in the neighborhood of dominant edges) in the other image. Accordingly, the second condition $RN_{2}$ is denoted as Equation (6) as follows:

(6) $${\mathrm{RN}}_{2}\in \{\begin{array}{cc} 1, & \mathrm{if}\;X_{ref}^{h}-\alpha X_{sen}^{h}\geq T_{2}\\ 0, & \mathrm{otherwise} \end{array}$$

where $T_{2}$ is a threshold value that controls the sensitivity to the amount of misregistration. $\alpha =\sigma \left(X_{ref}^{h}\right)/\sigma \left(X_{sen}^{h}\right)$ is the normalization factor. The final RN map is identified as simultaneously satisfying Equations (5) and (6).

#### 2.2.2. Extraction of CPs for the Reference Image

Theoretically, a large number of CPs should be extracted in regions where the misalignments are severe, in order to minimize such misalignments. In contrast, less CPs are sufficient for well-aligned regions. Based on this concept, local regions associated with the CPs distribution of the reference image are defined. Here, we use a quadtree-based segmentation approach to construct local regions by decomposing the reference image recursively into four equally sized regions. The approach begins with the entire reference image as a single region, after which the image is divided into four sub-regions according to a splitting criterion. Considering the number of extracted RN pixels in a local region compared with those in the entire image, the splitting criterion Q(s) can be defined as Equation (7) as follows:

(7) $$Q\left(s\right)=\{\begin{array}{cc} \mathrm{if}\;\frac{\sum_{r=1}^{w_{r}}\sum_{c=1}^{w_{c}}{\mathrm{RN}}_{r,c}}{\sum_{r=1}^{w_{r}}\sum_{c=1}^{w_{c}}X_{ref}}\geq \frac{\sum_{i=1}^{I}\sum_{j=1}^{J}{\mathrm{RN}}_{i,j}}{\sum_{i=1}^{I}\sum_{j=1}^{J}X_{ref}}, & \mathrm{split}\\ \mathrm{otherwise}, & \mathrm{stop} \end{array}$$

where $X_{ref}$ is the entire reference image, whose size is I×J; and (r,c) and (i,j) are the pixels in a given local region, whose size is $w_{r}\times w_{c}$, and the reference image $X_{ref}$, respectively. According to Equation (7), the left and right terms of the numerator denote the number of the RN pixels in the given local region and $X_{ref}$, and those of the denominator denote the number of all pixels in the given local region and $X_{ref}$, respectively. If the condition Q(s) is satisfied, the corresponding region is subdivided into four equally sized quadrant regions. This process proceeds until the region reaches the predefined minimal region size. From Equation (7), regions having a relatively large number of RN pixels, when compared with the number of RN pixels extracted over the entire image, are recursively subdivided. After finishing the segmentation, centroids of each sub-region are selected as CPs for the reference image. The CPs for the sensed image are at a position according to the consideration of the locally extracted RN amounts, as explained in the following sub-section.

#### 2.2.3. RN-Based Image Matching

RN-based image matching is performed by computing the number of extracted RN pixels within a locally defined region via a quadtree-based segmentation approach. To this end, we propose the RNCC approach, denoted as Equation (8) as follows:

(8) $$\mathrm{RNCC}=\overset{w_{r}}{\underset{r=1}{\sum }}\overset{w_{c}}{\underset{c=1}{\sum }}{\mathrm{RN}}_{r,c}$$

where $RN_{r,c}$ is whether an RN pixel is extracted, or not, at a pixel (r,c) in a given segment, whose size is $w_{r}\times w_{c}$. The RNCC within a predefined search space is calculated, and the pixel position ($r_{min},c_{min}$) where the extracted RN pixels are at a minimum is determined as the position of the CPs for the sensed image. Let us assume that a number of S segments are constructed over the entire overlapping region between the multi-temporal images. For each CP $c_{1}^{s}\left(s=1,\ldots ,S\right)$ in the reference image, a location of CP $c_{2}^{s}\left(s=1,\ldots ,S\right)$ in the sensed image is determined using Equation (8) accordingly.

After extracting all of the CPs from each segment, we employ a piecewise linear function, which is known to be appropriate for mitigating local distortions between VHR images. The piecewise linear function divides $X_{ref}$ into triangular regions using Delaunay’s triangulation algorithm, using CPs as the vertexes of the triangles. When the CPs in $X_{ref}$ are triangulated, the correspondences in $X_{sen}$ are triangulated accordingly. Then, each triangulated region in $X_{sen}$ is mapped to the corresponding region of $X_{ref}$ via affine transformation.

## 3. Experiments and Results

### 3.1. Dataset Construction

Test data are constructed from five Kompsat-3 images, each of which covers approximately 16.8 × 15 km over Daejeon in South Korea with a 0.7-m spatial resolution. The processing level is 1R and is radiometrically corrected. Kompsat-3 images have different incidence and azimuth angles that might cause geometric misalignments among them. The test scenes include various land covers, such as urban, agriculture, mountains, etc. First, the georegistration of the Kompsat-3 images to the map coordinates is conducted, and then all of the possible combinations of the georegistered Kompsat-3 pairs are considered to apply the fine co-registration approach. The detailed specifications of the scene are listed in Table 1.

### 3.2. Global Georegistration Results

**Figure 3:**
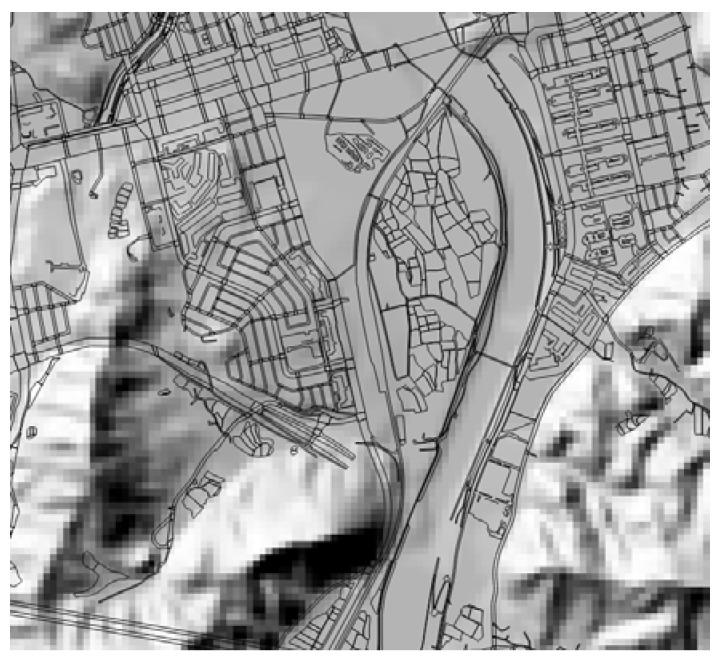
An example of a selected map layer and generated DEM.

For reference data, 1:5000 scale topographic maps were downloaded from the official website of the National Geographic Information Institute of Korea. Among the eight different map layer categories, we selected the road and land use layers for matching purposes, while the contour layers were extracted for DEM generation. Figure 3 shows an example of the selected map layers on the generated DEM.

**Figure 4:**
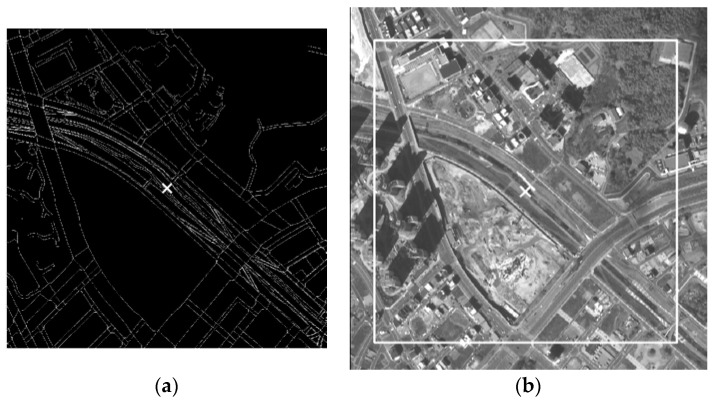
An RECC window matching (computed $CV_{4}$: 1.2 pixels—average distance between the maximal RECC location and the four locations with largest RECC in (**b**)). (**a**) Rasterized map layer and (**b**) matched location on a VHR image.

The selected map layers, in vector form, are projected into a VHR image space and rasterized as one sample window of 500 × 500 pixels, as shown in Figure 4a. The window slides over the image region in Figure 4b to compute RECC and $CV_{4}$ for the matched location. The marker and the box in Figure 4b show the location and the window boundary.

**Figure 5:**
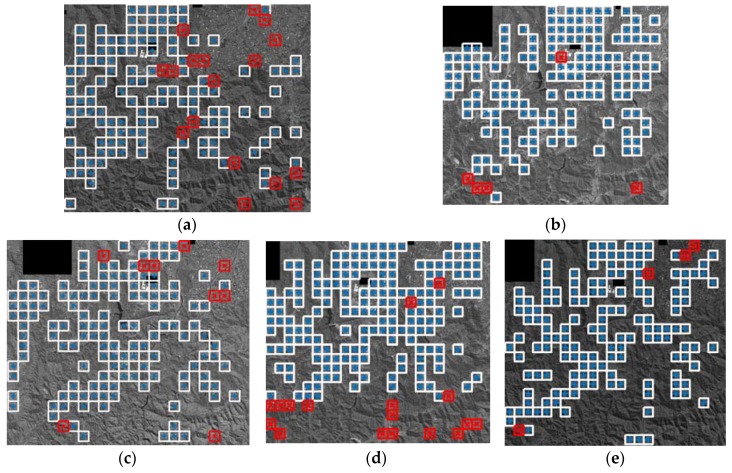
RECC matching results: (**a**) K3-1; (**b**) K3-2; (**c**) K3-3; (**d**) K3-4; and (**e**) K3-5. Correct matches and outliers are marked as blue + and red x, respectively.

RECC window matching is conducted for the entire VHR image region, except for the very large black region that is missing because of a security issue. Figure 5 shows the distribution of the matched locations. The matched points are distributed over the entire image space, while the lack of features in some image regions, such as high mountains, hindered the rich matching point extraction. Note that the local geometric errors are estimated at the matched locations and used for the bias-compensation modeling. Matching outliers are statistically detected during the bias-compensation model estimation and are marked as ‘X’ in the figure. The number of detected outliers is listed in Table 2. Table 2 also shows the bias-compensation precision before and after outlier removal. The matched points are used to model the affine model (Equation (3)) and the precision (fitness to the model) was at an approximately one-pixel level.

**Figure 6:**
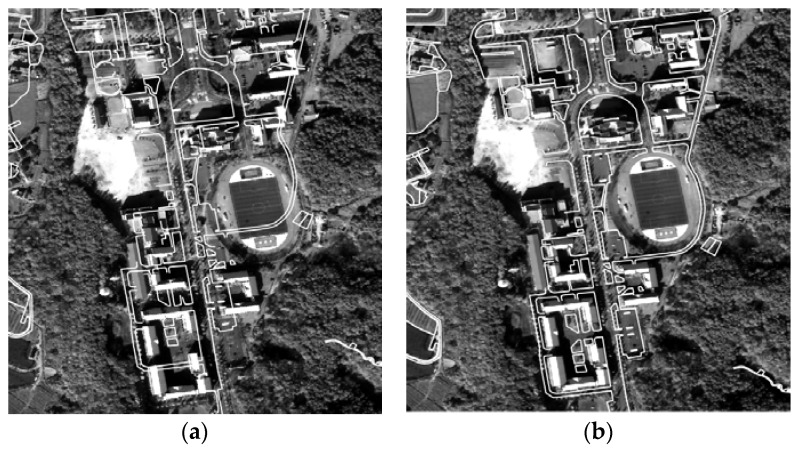
Overlay of map layers and VHR image: (**a**) before and (**b**) after the bias-compensation.

Figure 6 shows the overlay of map layers with the VHR image before and after the global georegistration in which the accuracy improvement can be observed.

### 3.3. Fine Co-Registration Results

As the RN map derived from the VHR multi-temporal images will include noise pixels because of the spatial heterogeneity in the pixels, we first generate pyramid images that resample the image at a reduced size. Applying the RN extraction approach on the pyramid images allows for extraction of reliable RN pixels, as well as improvement of efficiency. The pyramid images were reduced by four times when compared with the original images. The values of σ and k that control the thickness of the edges were set to 1.6 and 2, respectively, which are the values recommended in Han et al. [29]. Two thresholds, $T_{1}$ and $T_{2}$, that are related to the RN extraction were automatically selected by applying an expectation maximization method [30]. The minimal and maximal sizes of the quadtree structure for the segmentation were set to 256 and 1024, respectively. The size of the searching space for finding the CPs for the sensed image was set to a range within 12 pixels (i.e., approximately 8.4 m, according to the spatial resolution of Kompsat-3), which is a relatively limited size owing to the impact of the global georegistration results.

**Figure 7:**
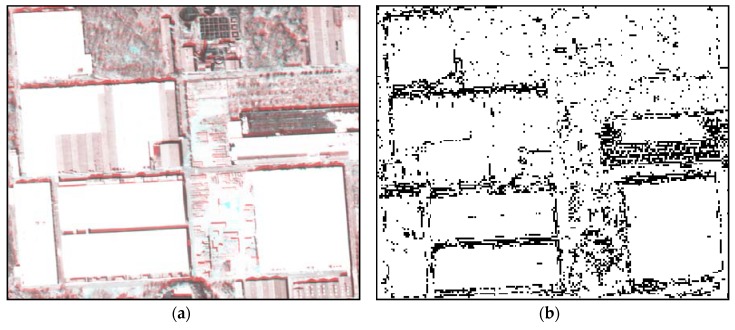
An example of an RN extraction result: (**a**) georegistered multi-temporal images and (**b**) extracted RN from the images.

An example of the extracted RN is presented in Figure 7 with a corresponding region over the georegistered multi-temporal images. Figure 7a shows the multi-temporal images with false-color composition of the reference (K3-1) and sensed (K3-2) images. The figure shows a local misalignment, which is expressed by the red color along the boundaries of objects. The extracted RN pixels, which are represented by black pixels in Figure 7b, have a tendency to correspond properly with the local misalignment occurring between the multi-temporal images.

**Figure 8:**
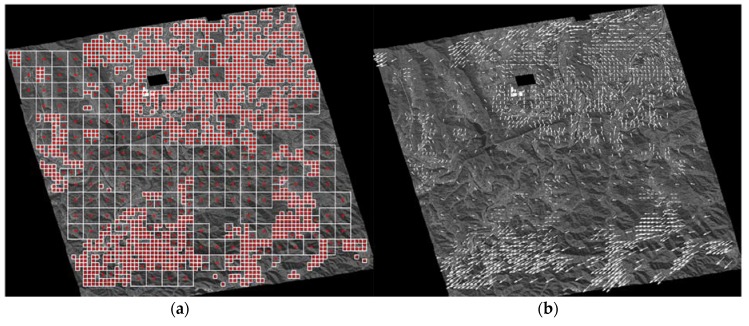
Extracted corresponding points (CPs) from the K3-1 and K3-2 image pair. (**a**) Determined segments are expressed as white rectangles, and corresponding CPs, which are in the centroids of the segments, are marked as red points; (**b**) Location differences between CPs of the reference and sensed images are indicated as arrows.

The location of the CPs is determined by RNCC. Because of the limited length of this paper, only the extracted CPs from the K3-1 and K3-2 image pair are presented in Figure 8. The CPs are marked as red points with constructed corresponding segments, which are represented as white rectangles in Figure 8a. As one can see, the extracted CPs were evenly distributed over the entire scene, and a particularly larger number of CPs was extracted in built-up regions where a local misalignment was severe in general. Location differences between the CPs extracted from the reference and sensed images are indicated by arrows in Figure 8b.

**Figure 9:**
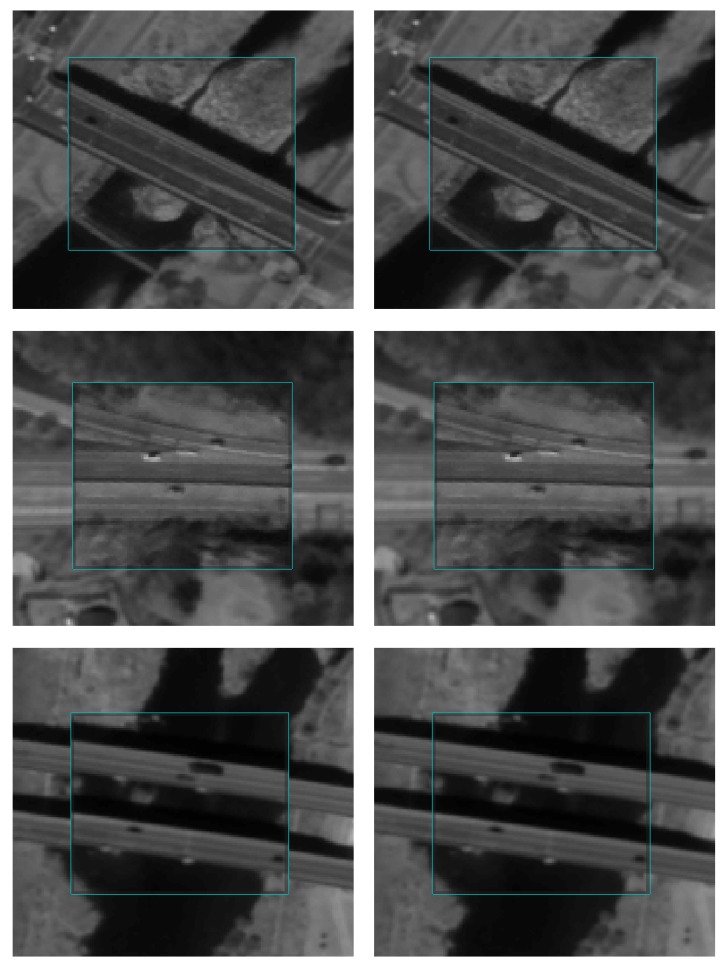

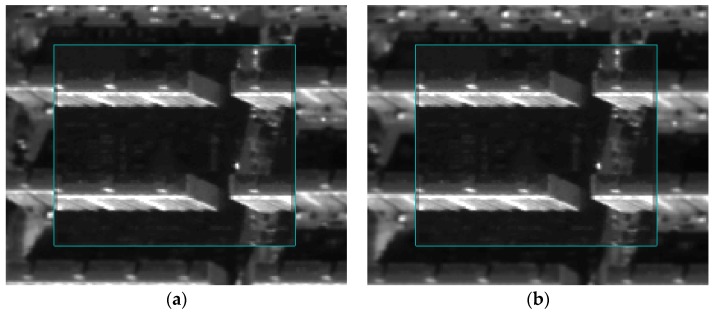
Visual inspection of geo/co-registration results: (**a**) georegistration results and (**b**) co-registration results.

**Figure 10:**
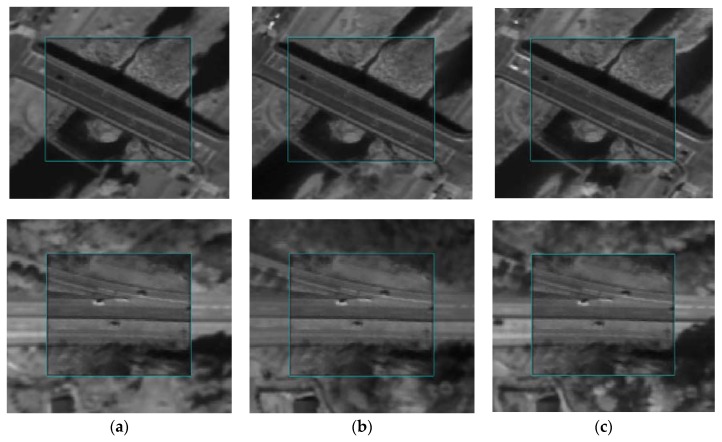
Visual inspection of co-registration results focusing on multi-temporal images: (**a**) K3-1 and K3-3, (**b**) K3-1 and K3-4, and (**c**) K3-1 and K3-5.

For visual assessment, georegistration and co-registration results were compared as shown in Figure 9. The inside rectangles are captured from the reference image (K3-1), and the outside rectangles are captured from the sensed image (K3-2), transformed by the RECC-based georegistration (Figure 9a) and the RNCC-based co-registration (Figure 9b), respectively. The co-registration performance can thus be compared while checking the boundaries of the two rectangles (which are highlighted in cyan) and whether the lines or objects along the boundaries are properly aligned. As one can see, applying the co-registration approach improves the alignment when compared with those in which it is not applied. To evaluate the co-registration performance from a time-series point of view, a visual inspection regarding the co-registration results between the multi-temporal image pairs was conducted, and some of the same regions in Figure 9 are presented in Figure 10 (i.e., multi-temporal image pairs between K3-1 and K3-3, K3-1 and K3-4, and K3-1 and K3-5 are presented in Figure 10a–c, respectively). Regardless of the image pairs, they showed reliable geometric alignments along the rectangles.

To perform a quantitative analysis to confirm the superiority of the co-registration performance, correlation coefficient (CC) values were calculated between the reference and sensed images. The CC values were calculated from all combinations of the tested image pairs. The calculated results are presented in Table 3, in which one can see that the proposed co-registration approach effectively improved the CC values in all cases because of the properly constructed piecewise linear function with a large number of CPs. A student t-test for the CC improvement between RECC and RNCC was performed. We confirmed that the null hypothesis was rejected at a 99% confidence level, which means that the level of improvement is statistically significant.

## 4. Conclusions

This study proposed an automated geo/co-registration framework for full-scene images acquired from a VHR optical satellite sensor. The proposed method first globally georegistered the images based on RECC with a reference map, and then a fine co-registration process was conducted based on RNCC between the georegistered images. Experiments on georegistration implemented using five Kompsat-3 full scenes showed RPC bias-compensation precision of approximately one-pixel level. Furthermore, experiments regarding co-registration could statistically show significant improvement in geometric alignment while reducing local misalignments between all combinations of multi-temporal image pairs. In a future study, VHR images acquired from multi-sensors, such as GeoEye and WorldView, will be tested to prove the robustness of the proposed approach.

## Figures and Tables

**Table 1:** Specifications of tested Kompsat-3 images.

Sensor	Kompsat-3				
Scene ID	K3-1	K3-2	K3-3	K3-4	K3-5
Spatial resolution	0.7 m	0.7 m	0.7 m	0.7 m	0.7 m
Processing level	1R	1R	1R	1R	1R
Acquisition date	7 February 2016	25 March 2015	23 October 2014	3 March 2014	16 November 2013
Incidence/azimuth	38.0°/196.8°	23.2°/125.4°	25.6°/238.1°	12.1°/146.4°	10.1°/260.6°
Size (line ×sample)	20,452 × 24,060 pixels	21,280 × 24,060 pixels	20,464 × 24,060 pixels	21,740 × 24,060 pixels	22,376 × 24,060 pixels

**Table 2:** Rational polynomial coefficients (RPC) bias-compensation precision before and after outlier removal.

Scene ID	Number of Outliers [Points]	Bias Precision before Outlier Removal (col/row) [Pixels]	Bias Precision after Outlier Removal (col/row) [Pixels]
K3-1	17	1.89/5.01	0.51/0.59
K3-2	5	2.50/2.81	1.90/1.68
K3-3	9	0.90/0.86	0.75/0.74
K3-4	17	2.86/3.27	1.21/0.99
K3-5	4	1.16/1.93	0.81/0.93

**Table 3:** Accuracy assessment of georegistration and co-registration results. RECC—relative edge cross-correlation; RNCC—registration noise-based cross-correlation.

Reference Image	Sensed Image	Number of Corresponding Points	Correlation Coefficient (RECC)	Correlation Coefficient (RNCC)
K3-1	K3-2	2455	0.757	0.799
	K3-3	1591	0.583	0.625
	K3-4	3014	0.774	0.818
	K3-5	3095	0.687	0.734
K3-2	K3-3	665	0.539	0.591
	K3-4	1159	0.841	0.851
	K3-5	617	0.630	0.663
K3-3	K3-4	1831	0.622	0.664
	K3-5	1323	0.710	0.742
K3-4	K3-5	1772	0.757	0.785

## References

[1] Zhao Y. (2014). Crop growth dynamics modeling using time-series satellite imagery. Land Surf. Remote Sens. II.

[2] Mennis J., Viger R. Analyzing time series of satellite imagery using temporal map algebra. Proceedings of the ASPRS 2004 Annual Convention.

[3] Laneve G., Cadau E.G., De Rosa D. Change detection analysis on time series of satellite images with variable illumination conditions and spatial resolution. Proceedings of the MultiTemp 2007. International Workshop on the Analysis of Multi-temporal Remote Sensing Images.

[4] Yang X., Lo C.P. (2002). Using a time series of satellite imagery to detect land use and land cover changes in the Atlanta, Georgia metropolitan area. Int. J. Remote Sens..

[5] Stumpf A., Malet J.P., Delacourt C. (2017). Correlation of satellite image time-series for the detection and monitoring of slow-moving landslides. Remote Sens. Environ..

[6] Behling R., Roessner S., Segl K., Kleinschmit B., Kaufmann H. (2014). Robust automated image co-registration of optical multi-sensor time series data: Database generation for multi-temporal landslide detection. Remote Sens..

[7] Aguilar M.A., del Mar Saldana M., Aguilar F.J. (2013). Assessing geometric accuracy of the orthorectification process from GeoEye-1 and WorldView-2 panchromatic images. Int. J. Appl. Earth Obs..

[8] Oh J., Lee C. (2015). Automated bias-compensation of rational polynomial coefficients of high resolution satellite imagery based on topographic maps. ISPRS J. Photogramm. Remote Sens..

[9] Teo T.A. (2011). Bias compensation in a rigorous sensor model and rational function model for high-resolution satellite images. Photogramm. Eng. Remote Sens..

[10] Shen X., Li Q., Wu G., Zhu J. (2017). Bias compensation for rational polynomial coefficients of high-resolution satellite imagery by local polynomial modeling. Remote Sens..

[11] Konugurthi P.K., Kune R., Nooka R., Sarma V. (2016). Autonomous ortho-rectification of very high resolution imagery using SIFT and genetic algorithm. Photogramm. Eng. Remote Sens..

[12] Fraser C.S., Hanley H.B. (2005). Bias-compensated RPCs for sensor orientation of high-resolution satellite imagery. Photogramm. Eng. Remote Sens..

[13] Oh J., Lee C., Seo D.C. (2013). Automated HRSI georegistration using orthoimage and SRTM: Focusing KOMPSAT-2 imagery. Comput Geosci..

[14] Pan H., Tao C., Zou Z. (2016). Precise georeferencing using the rigorous sensor model and rational function model for ZiYuan-3 strip scenes with minimum control. ISPRS J. Photogramm. Remote Sens..

[15] Zitová B., Flusser J. (2003). Image registration methods: A survey. Image Vis. Comput..

[16] Huo C., Pan C., Huo L., Zhou Z. (2012). Multilevel SIFT matching for large-size VHR image registration. IEEE Geosci. Remote Sens. Lett..

[17] Yu L., Zhang D., Holden E.J. (2008). A fast and fully automatic registration approach based on point features for multi-source remote-sensing images. Comput. Geosci..

[18] Lowe D.G. (2004). Distinctive image features from scale-invariant keypoints. Int. J. Comput. Vis..

[19] Bay H., Ess A., Tuytelaars T., Van Gool L. (2008). Speeded-up robust features (SURF). Comput. Vis. Image Underst..

[20] Oh J., Lee C., Eo Y., Bethel J. (2012). Automated georegistration of high-resolution satellite imagery using a RPC model with airborne lidar information. Photogramm. Eng. Remote Sens..

[21] Han Y., Bovolo F., Bruzzone L. (2015). An approach to fine coregistration between very high resolution multispectral images based on registration noise distribution. IEEE Trans. Geosci. Remote Sens..

[22] Hong G., Zhang Y. (2008). Wavelet-based image registration technique for high-resolution remote sensing images. Comput. Geosci..

[23] Arévalo V., González J. (2008). Improving piecewise linear registration of high-resolution satellite images through mesh optimization. IEEE Trans. Geosci. Remote Sens..

[24] Han Y., Choi J., Byun Y., Kim Y. (2014). Parameter optimization for the extraction of matching points between high-resolution multisensory images in urban areas. IEEE Trans. Geosci. Remote Sens..

[25] Ye Y., Shan J. (2014). A local descriptor based registration method for multispectral remote sensing images with non-linear intensity differences. ISPRS J. Photogramm. Remote Sens..

[26] Han Y., Bovolo F., Bruzzone L. (2017). Segmentation-based fine registration of very high resolution multitemporal images. IEEE Trans. Geosci. Remote Sens..

[27] Habib A., Han Y., Xiong W., He F., Zhang Z., Crawford M. (2016). Automated ortho-rectification of UAV-based hyperspectral data over an agricultural field using frame RGB imagery. Remote Sens..

[28] Aicardi I., Nex F., Gerke M., Lingua A.M. (2016). An Image-Based Approach for the Co-Registration of Multi-Temporal UAV Image Datasets. Remote Sens..

[29] Han Y., Bovolo F., Bruzzone L. (2016). Edge-based registration-noise estimation in VHR multitemporal and multisensor images. IEEE Geosci. Remote Sens. Lett..

[30] Carson C., Belongie S., Greenspan H., Malik J. (2002). Blobworld: Image segmentation using expectation-maximization and its application to image querying. IEEE Trans. Pattern Anal. Mach. Intell..

